# Prognostic Utility of Calcium Scoring as an Adjunct to Stress Myocardial Perfusion Scintigraphy in End-Stage Renal Disease

**DOI:** 10.1016/j.amjcard.2016.02.003

**Published:** 2016-05-01

**Authors:** William E. Moody, Erica L.S. Lin, Matthew Stoodley, David McNulty, Louise E. Thomson, Daniel S. Berman, Nicola C. Edwards, Benjamin Holloway, Charles J. Ferro, Jonathan N. Townend, Richard P. Steeds, Birmingham Cardio-Renal Group

**Affiliations:** a Birmingham Cardio-Renal Group, Department of Cardiology, Institute of Cardiovascular Sciences, Nuffield House, Queen Elizabeth Hospital Birmingham, Edgbaston; b Department of Cardiac Imaging and Nuclear Cardiology, S. Mark Taper Foundation Imaging Center Los Angeles, California

## Abstract

Coronary artery calcium score (CACS) is a strong predictor of adverse cardiovascular events in the general population. Recent data confirm the prognostic utility of single-photon emission computed tomographic (SPECT) imaging in end-stage renal disease, but whether performing CACS as part of hybrid imaging improves risk prediction in this population is unclear. Consecutive patients (n = 284) were identified after referral to a university hospital for cardiovascular risk stratification in assessment for renal transplantation. Participants underwent technetium-99m SPECT imaging after exercise or standard adenosine stress in those unable to achieve 85% maximal heart rate; multislice CACS was also performed (Siemens Symbia T16, Siemens, Erlangen, Germany). Subjects with known coronary artery disease (n = 88) and those who underwent early revascularization (n = 2) were excluded. The primary outcome was a composite of death or first myocardial infarction. An abnormal SPECT perfusion result was seen in 22% (43 of 194) of subjects, whereas 45% (87 of 194) had at least moderate CACS (>100 U). The frequency of abnormal perfusion (summed stress score ≥4) increased with increasing CACS severity (p = 0.049). There were a total of 15 events (8 deaths, and 7 myocardial infarctions) after a median duration of 18 months (maximum follow-up 3.4 years). Univariate analysis showed diabetes mellitus (Hazard ratio [HR] 3.30, 95% CI 1.14 to 9.54; p = 0.028), abnormal perfusion on SPECT (HR 5.32, 95% CI 1.84 to 15.35; p = 0.002), and moderate-to-severe CACS (HR 3.55, 95% CI 1.11 to 11.35; p = 0.032) were all associated with the primary outcome. In a multivariate model, abnormal perfusion on SPECT (HR 4.18, 95% CI 1.43 to 12.27; p = 0.009), but not moderate-to-severe CACS (HR 2.50, 95% CI 0.76 to 8.20; p = 0.130), independently predicted all-cause death or myocardial infarction. The prognostic value of CACS was not incremental to clinical and SPECT perfusion data (global chi-square change = 2.52, p = 0.112). In conclusion, a perfusion defect on SPECT is an independent predictor of adverse outcome in potential renal transplant candidates regardless of the CACS. The use of CACS as an adjunct to SPECT perfusion data does not provide incremental prognostic utility for the prediction of mortality and nonfatal myocardial infarction in end-stage renal disease.

Renal transplantation remains the most successful and cost-effective treatment for patients with end-stage renal disease (ESRD), significantly improving cardiovascular (CV) outcomes compared with maintenance dialysis.^1^ Even after transplantation, however, patients remain at high risk of long-term CV complications. To ensure that graft survival is not limited by premature CV death, both US and UK regulatory bodies recommend noninvasive CV assessment of those transplant candidates with multiple risk factors or diabetes, although there is no clear guidance on which imaging method to use.^2^, ^3^ The current suggestion is to adopt an imaging protocol for CV risk stratification according to “best local expertise.” Accordingly, many transplant centers continue to use stress myocardial perfusion scintigraphy because of longstanding data supporting its prognostic utility in ESRD.^4^, ^5^, ^6^, ^7^ Despite this, myocardial perfusion scintigraphy has poor positive predictive value for identifying coronary artery stenosis on invasive angiography.^5^, ^8^ Moreover, it is not able to detect subclinical atherosclerosis, potentially predisposing the patient in the longer term to subsequent obstructive CV events. Hybrid single-photon emission computed tomographic (SPECT)/CT imaging offers an attractive opportunity to combine anatomic measures of coronary artery calcification alongside a functional assessment of myocardial ischemia. Coronary artery calcium score (CACS) is a surrogate marker of atherosclerotic burden and a strong predictor of adverse CV events in subjects at intermediate risk from the general population.^9^ The predictive role of CACS in subjects with ESRD, however, is less certain.^10^, ^11^ Despite the very high burden of coronary calcification in this population, there is only a modest association between CACS and perfusion defects.^12^, ^13^ In the present study, we hypothesize that CACS will provide an incremental benefit for the prediction of death and first myocardial infarction (MI) in patients with ESRD beyond that provided by perfusion defect scores on myocardial perfusion imaging.

## Methods

**Figure 1:**
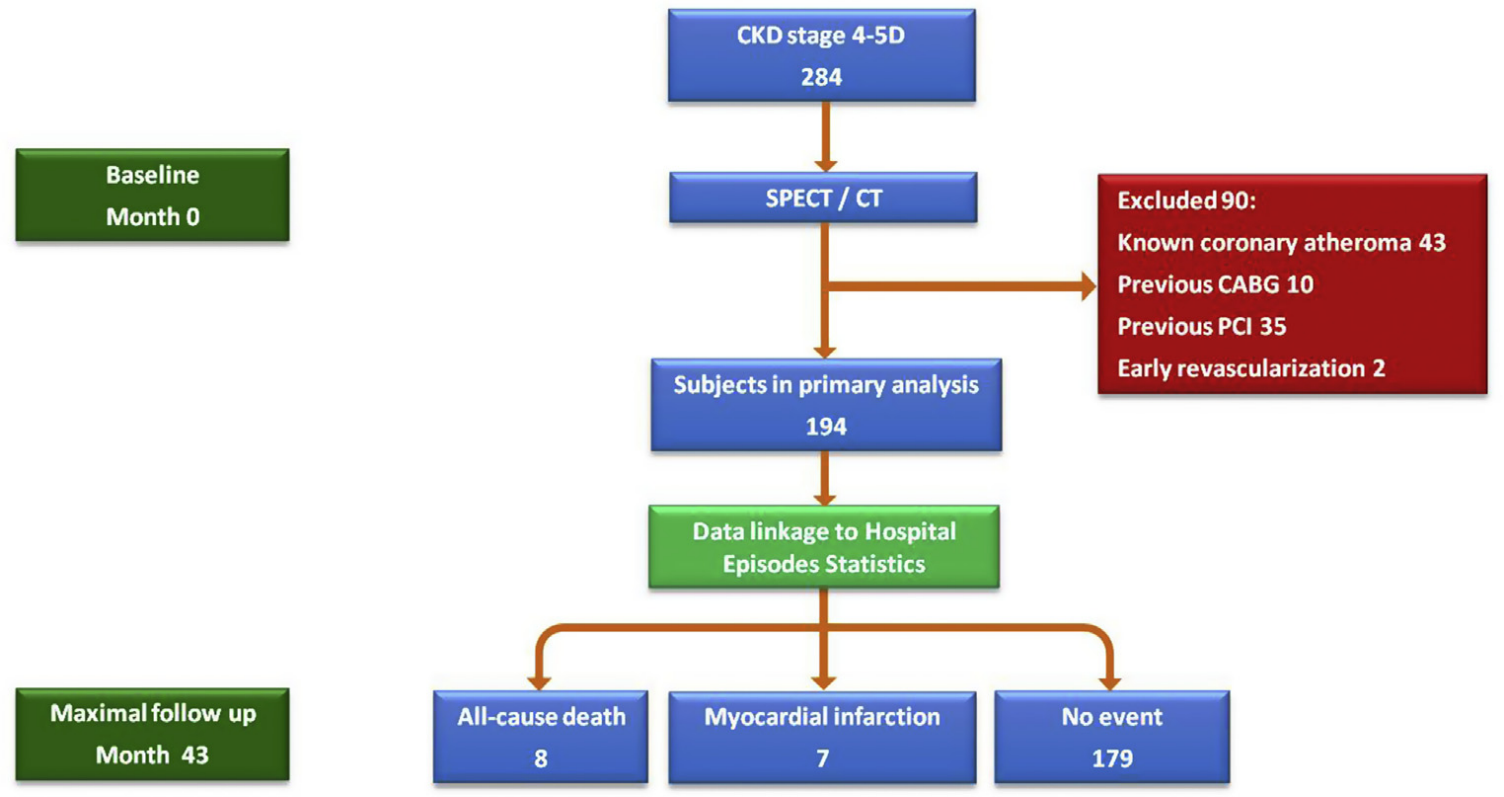
Study consort diagram. ^∗^Six of 88 subjects (7%) excluded from the analysis because of previous coronary atheroma, PCI, or CABG underwent early revascularization. Two further subjects without a baseline diagnosis of coronary atheroma underwent early revascularization (1 percutaneous coronary intervention and 1 coronary artery bypass graft surgery) driven by the SPECT/CT result.

Consecutive patients with chronic kidney disease (CKD) stage 4 to 5D were identified after referral to Queen Elizabeth Hospital Birmingham for CV risk stratification as part of a pretransplant screening work-up from January 2011 to December 2013. In accordance with current guidelines compiled by a Joint Working Party of The British Transplantation Society and The Renal Association,^3^ subjects were referred for noninvasive CV risk assessment if they fulfilled any of the following criteria: age ≥50years, diabetes, suspected angina, or known ischemic heart disease. Those subjects with a history of MI, coronary atheroma or stenosis on angiography, or previous percutaneous or surgical revascularization were excluded from the present study (Figure 1). Formal ethical approval was not required because this study was a retrospective assessment of solely clinical data and was therefore regarded as a health outcomes evaluation. The conduct and reporting of this study was guided by the Strengthening the Reporting of Observational Studies in Epidemiology statement.^14^

Demographic and anthropometric data were collected on all patients through review of patient electronic records. In addition, a standard prescan assessment involving a detailed patient interview was performed to obtain information on symptoms, CV risk factors, previous CV events, and medication. A Duke pretest probability of coronary artery disease (CAD) was calculated at the time of the imaging study.^15^ Routine hematology and biochemistry at the time of the test were also recorded. Diabetes mellitus (DM) was defined as a fasting glucose >126 mg/dl, history of DM, diabetic nephropathy, or currently receiving hypoglycemic treatment. Hypertension was defined as an office blood pressure >140/90 mm Hg or currently taking antihypertensive medication. Hypercholesterolemia was defined as a serum cholesterol of >193 mg/dl or currently taking lipid reduction therapy. A history of CV disease was defined as having any of the following: CAD (MI, previous percutaneous, or surgical revascularization), heart failure, stroke, and peripheral vascular disease. Significant family history of CV disease was defined as a first degree relative with a history of MI or ischemic stroke in men younger than 55 years and in women younger than 65 years.

Patients were asked to discontinue β blockers, rate-limiting calcium channel blockers, and caffeine products 24 hours before testing, and nitrate compounds were discontinued >6 hours before testing. All participants underwent 2-day stress-rest technetium-99m SPECT imaging with exercise treadmill or standard adenosine stress (140 μg/kg/min for 6 minutes) in those unable to achieve 85% maximal heart rate; and multislice CACS was performed as routine. CT-based attenuation correction was performed in all patients during reconstruction of the SPECT data (Symbia T16, Siemens, Erlangen, Germany).

SPECT myocardial perfusion images were visually analyzed by 2 experienced observers (RPS and BH) blinded to outcome variables (Quantitative Perfusion SPECT; Hermes Medical Solutions, Stockholm, Sweden). In addition to examination of raw images in cine mode, both nonattenuated and attenuated images were reviewed, and a report produced consistent with recommendations outlined in the American Society of Nuclear Cardiology Imaging Guidelines for Nuclear Cardiology Procedures.^16^ Short-axis and vertical long-axis tomograms were divided into 17 segments for each study,^16^ and segmental tracer uptake was evaluated using a validated semiquantitative 5-point scoring system (0, normal; 1, equivocal; 2, moderate; 3, severe reduction of radioisotope uptake; and 4, absence of detectable tracer uptake).^17^ The summed stress and rest scores were obtained by adding the scores of the 17 segments of the respective images. The sum of the differences between each of the 17 segments from these images was defined as the summed difference score, representing the amount of ischemia. These indexes were converted to the percentage of total myocardium involved with stress, ischemic, or fixed defects by dividing the summed scores by 68 (the maximum potential score = 4 × 17) and multiplying by 100. The presence of abnormal perfusion was defined as a summed stress score of 4 or greater.^18^ A stress-induced total perfusion defect size (PDS) >15% or an ischemic PDS >10% defined high risk for cardiac events.^19^ Cardiac volumes and left ventricular (LV) ejection fraction were also calculated from the gated SPECT images.

The CACS was calculated according to Agatston et al^20^ by the same 2 independent observers blinded again to outcome data. Lesions were manually traced on CT images before semiautomatic quantification-derived vessel-specific scores were summated to yield the total CACS (syngo.via; Leonardo; Siemens Medical Solutions, Forchheim, Germany). Minimal, mild, moderate, and severe coronary calcification were defined as Agatston scores of 0 to 10 U, 11 to 100 U, 101 to 400 U, and >400 U, respectively.^19^

The primary outcome was a composite of all-cause death or MI. Myocardial infarction was defined as a clinical (or pathologic) event caused by myocardial ischemia where there is evidence of myocardial injury or necrosis as defined by an increase and/or decrease of cardiac biomarkers in the presence of typical symptoms or electrocardiographic changes, or imaging evidence of new loss of viable myocardium or new regional wall motion abnormality.^21^ Patients who had revascularization within 90 days of the imaging study were identified and excluded from the analysis to avoid inclusion of outcomes that may have been driven temporally by the SPECT/CT result.^6^ The event of all-cause death was examined separately as a secondary outcome. Patients who underwent renal transplant surgery during the study period were also identified.

Patient follow-up data were retrieved by an observer blinded to the clinical and imaging data (WEM). Every patient in the National Health Service has a unique identifier which enables outcomes to be tracked using the Hospital Episodes Statistics (HES) database, an administrative data warehouse containing admissions to all National Health Service hospitals in England.^22^ It contains detailed records relating to individual patient treatments, with data extraction facilitated using codes on procedural classifications (*Office of Population Censuses and Surveys Classification of Interventions and Procedures, Fourth revision*) and medical classifications (*World Health Organization International Classification of Disease, Tenth revision*).^23^, ^24^ With regard to outcome analysis, HES data alone have the limitation of only capturing deaths occurring in a hospital setting. To obtain the complete mortality list, the study cohort was also cross-referenced with mortality data from the Office for National Statistics, which collects information on all registered deaths in the UK. All outcomes were further verified by cross-referencing with individual hospital case notes held electronically.

Statistical analyses were performed with Stata, version 12 (StataCorp LP, College Station, Texas) and SAS (Statistical Analysis System, SAS Institute Inc., Cary, North Carolina). Data are expressed as mean ± SD, median (interquartile range), or frequency (%), unless otherwise stated. The normality of distribution for continuous variables was determined using normality plots and the Kolmogorov–Smirnov test. Baseline characteristics of the population were examined by CACS category and SPECT results. The Kruskal–Wallis analysis of variance was used to identify significant differences in central tendencies of continuously scaled variables between groups. Contingency table analysis was performed using the chi-square or Fisher's exact tests where appropriate.

Annualized event rates are expressed as the number of patients having first MI or all-cause death as a proportion of the number of patients at risk divided by the number of patient-years follow-up. Kaplan–Meier analysis of outcomes were based on discrete CACS categories (0 to 10, 11 to 100, 101 to 400, and >400 U) and SPECT categories (normal, total LV PDS dichotomized at 15%, ischemic PDS dichotomized at 10%). The date of the imaging test was used as time zero. In view of the beneficial CV effects of renal transplantation, those patients undergoing renal transplantation were censored at the time of the procedure.^25^ Two-sided log-rank tests were used to determine significance. Univariate and multivariate Cox proportional hazards models were used to identify the association between time-to-event and baseline clinical characteristics, SPECT and CACS results. Multivariate Cox regression analyses were also repeated using follow-up data not censored for transplantation. The change in the global chi-square statistic was calculated to determine the incremental prognostic value of clinical, SPECT, and CACS data. A p value <0.05 was considered statistically significant for all analyses.

## Results

In total, 284 consecutive patients (CKD stage 4 to 5D) with imaging performed from March 2011 to December 2013 were identified; of those, 88 had CAD at baseline. A further 2 subjects without a previous diagnosis of coronary atheroma underwent early revascularization (1 coronary artery bypass graft surgery and 1 percutaneous coronary intervention) after SPECT demonstrated a reversible PDS ≥10%, leaving 194 subjects available for inclusion in the present analysis (Figure 1).

The baseline characteristics of the study cohort are summarized in Table 1. Mean age was 56 years, 60% were men, 33% were diabetic, and 82% were hypertensive. Most patients were asymptomatic (75%). Two-thirds of patients had at least mild CACS (65%), and over a quarter had severe CACS (27%). In those with an abnormal SPECT result (28%), almost half (42%) had a total PDS ≥15% and a third (30%) had an ischemic PDS ≥10%.

Patients with a large total or ischemic PDS were older, less likely to be able to perform exercise treadmill stress and more likely to have accompanying LV dysfunction (Table 2). There was no difference in the mean number of cardiac risk factors between subjects with a normal SPECT result and those with a large perfusion defect.

As depicted in Table 3, subjects with a higher CACS were older and more frequently men and diabetic. There was a graded association between increasing CACS and worsening LV function. There was no significant association between the frequency of symptomatic chest pain and CACS severity.

**Figure 2:**
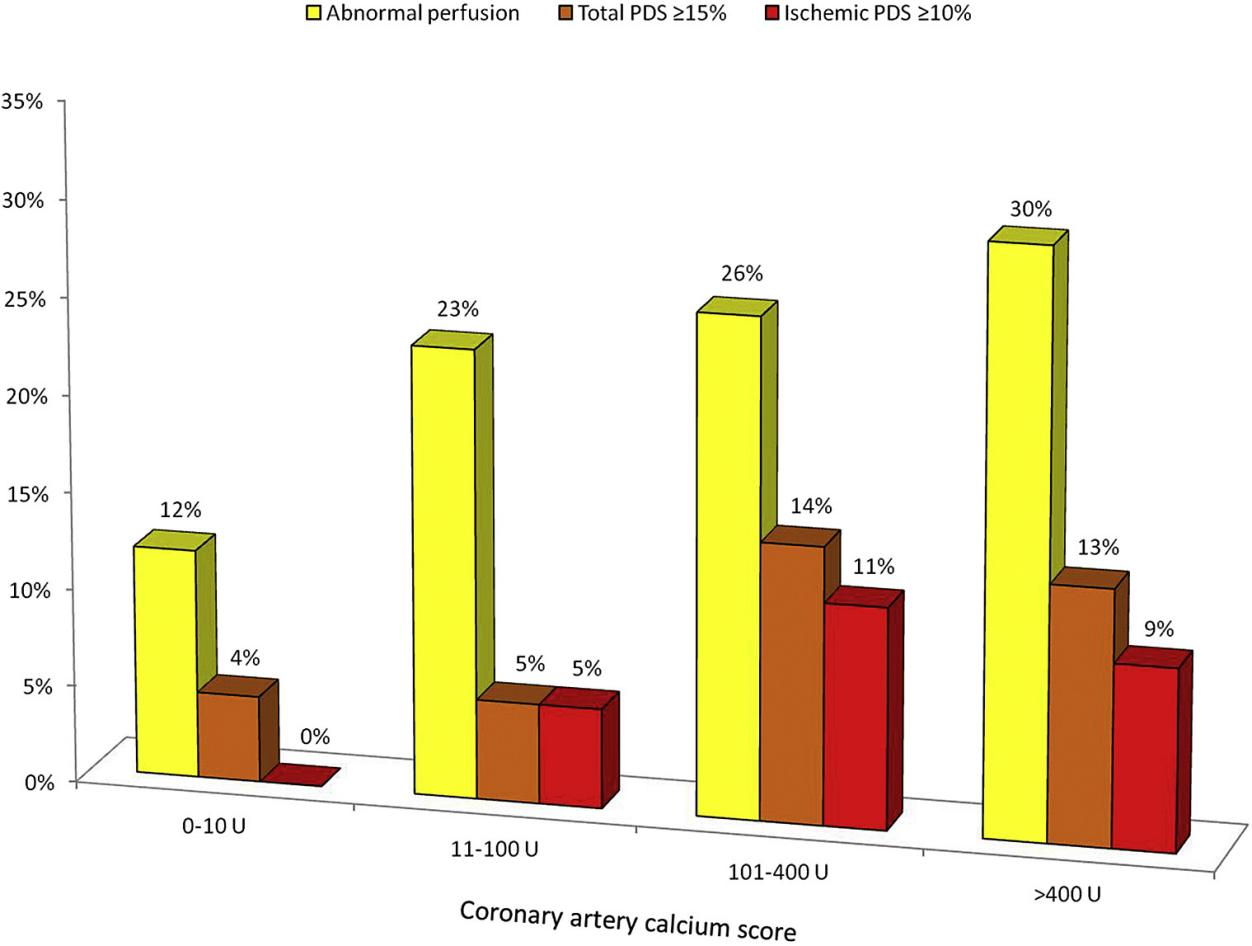
Relation between CACS and SPECT results. Relation between CACS severity and stress SPECT results (n = 194). The percentage of subjects with an abnormal SPECT result significantly increased with increasing CACS severity (p = 0.049). There was no significant association between the frequency of a large stress-induced total (>15%) or ischemic (>10%) LV perfusion defect and CACS severity. Twelve percent of subjects with minimal CACS (8 of 68) had abnormal perfusion on SPECT.

Subjects with a normal SPECT result had a lower median Agatston score compared to those with abnormal perfusion (35 U [IQR 0 to 349 U] vs 306 U [IQR 14 to 912 U]; p <0.01). There was a weak-graded association between the increasing proportion of patients with abnormal perfusion and increasing CACS severity (p = 0.049; Figure 2). There was, however, no significant association between CACS severity and the frequency of a large stress-induced total (≥10%) or ischemic (≥10%) PDS. An abnormal SPECT result was observed in 12% of subjects (8 of 68) with a CACS 0 to 10 and in 23% of subjects (9 of 39) with a CACS 11 to 100 U. In 4% of patients with only minimal CACS (3 of 68), a high-risk SPECT profile was demonstrated based on the stress-induced total PDS.

There were a total of 15 primary events (8 deaths and 7 MIs) after a median duration of 18 months (maximal follow-up 3.4 years). Forty-one patients (21%) underwent renal transplantation during the study period, one of whom died. This posttransplant death occurred 3 months after surgery in a subject with hypertension and type 2 DM; SPECT/CT imaging had demonstrated severe CACS (2,376 U) but no evidence of a perfusion defect. Two further patients who underwent transplant suffered a nonfatal MI, 1 subject with severe CACS >400 U and 1 subject with a detectable perfusion defect.

Univariate predictors of the primary outcome were DM, abnormal perfusion on SPECT, and an Agatston score of >100 U (all p <0.05; Table 4). In a multivariate model, abnormal perfusion on SPECT and diabetes, but not CACS independently predicted all-cause death/nonfatal MI. The results from multivariate Cox regression analyses performed using data not censored for transplantation showed no significant difference in the models shown (data not shown).

**Figure 3:**
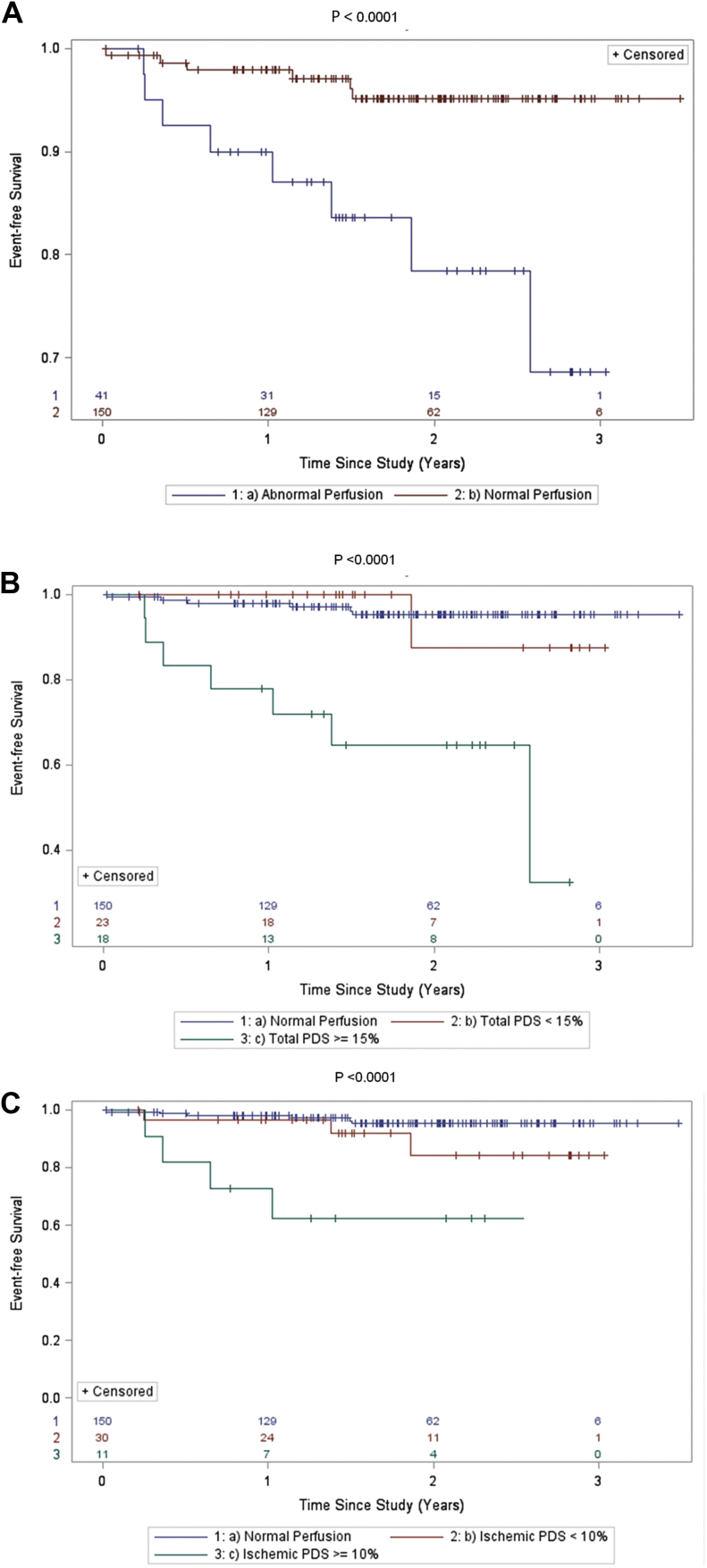
Kaplan–Meier curves comparing time to death or first MI according to stress SPECT results: *(A)* Perfusion abnormality; *(B)* total PDS; and *(C)* ischemic PDS. Two-sided log-rank tests were used to determine significance.

**Figure 4:**
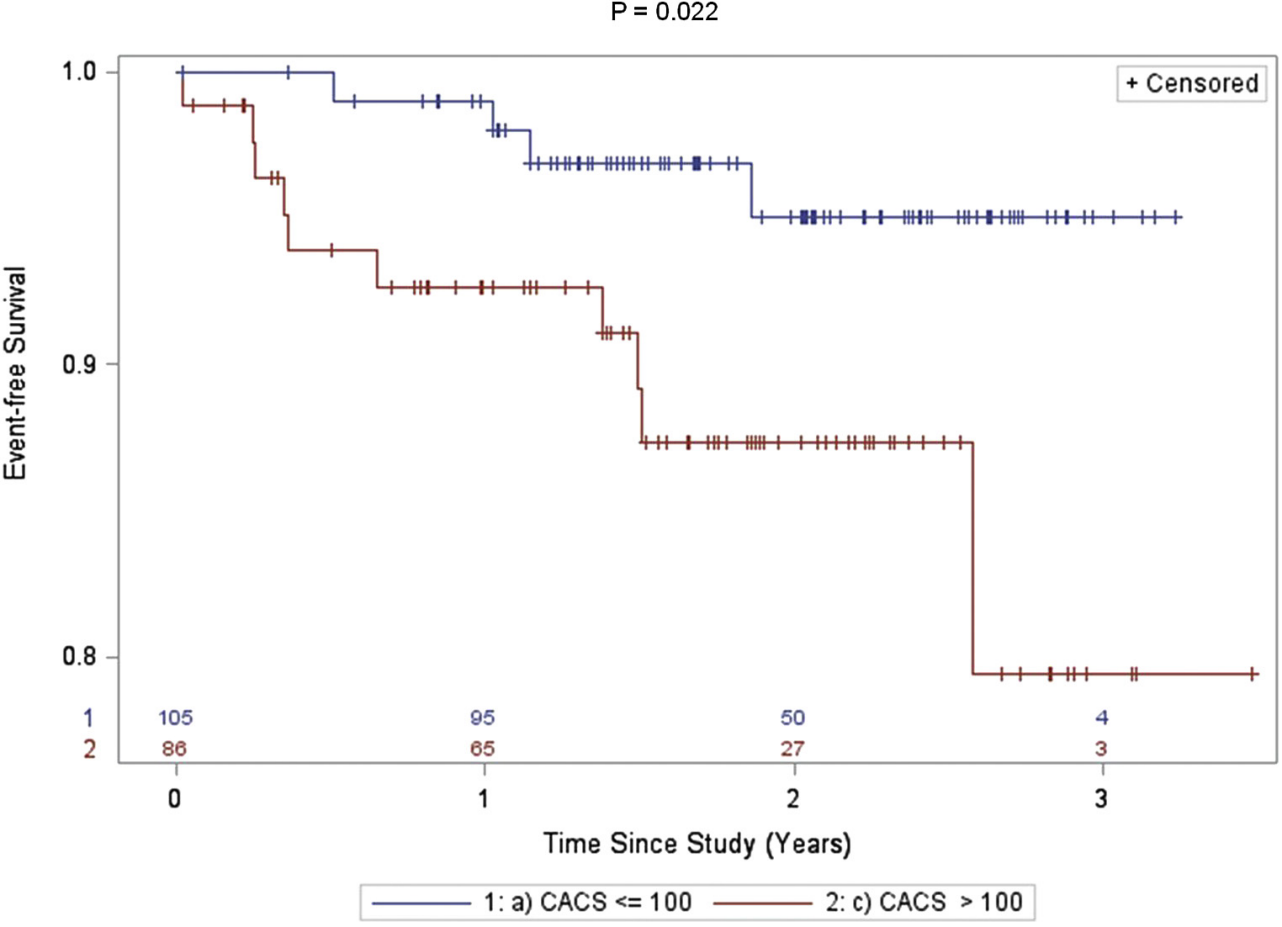
Kaplan–Meier curves comparing time to death or first MI according to the presence or absence of severe CACS. Two-sided log-rank tests were used to determine significance.

**Figure 5:**
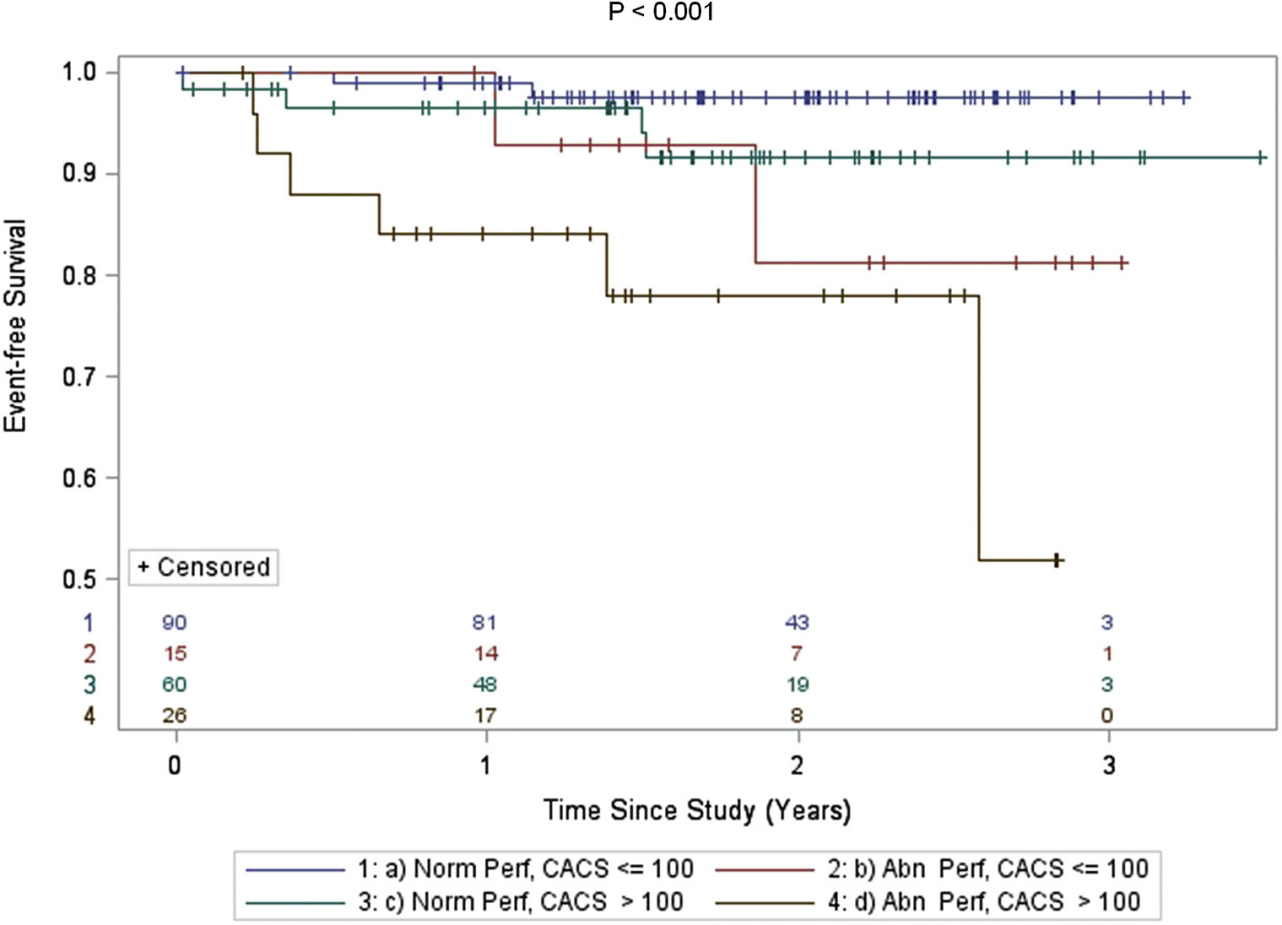
Kaplan–Meier curves comparing time to death or first MI according to integrated results of SPECT/CT. p Value shown corresponds to a significance difference between all 4 survival curves. There is also a significant difference in the survival curves for “abnormal perfusion/CACS ≤100” and “abnormal perfusion/CACS >100” (p <0.01). Two-sided log-rank tests were used to determine significance.

The risk for all-cause death/nonfatal MI increased significantly with the presence and extent of SPECT abnormality (Figure 3) and with the presence of moderate-to-severe CACS (Figure 4). In subjects with abnormal perfusion by SPECT (summed stress score ≥4), the annualized event rate for the primary outcome of all-cause death/nonfatal MI was 13.8% versus 2.8% in those with normal perfusion. Similarly, the incident rate of all-cause death/nonfatal MI was 12.8% for those subjects with moderate-to-severe CACS compared with 7.6% in those with a CACS <100 U. The value of integrating SPECT and CACS results for risk prediction is depicted in Figure 5.

**Figure 6:**
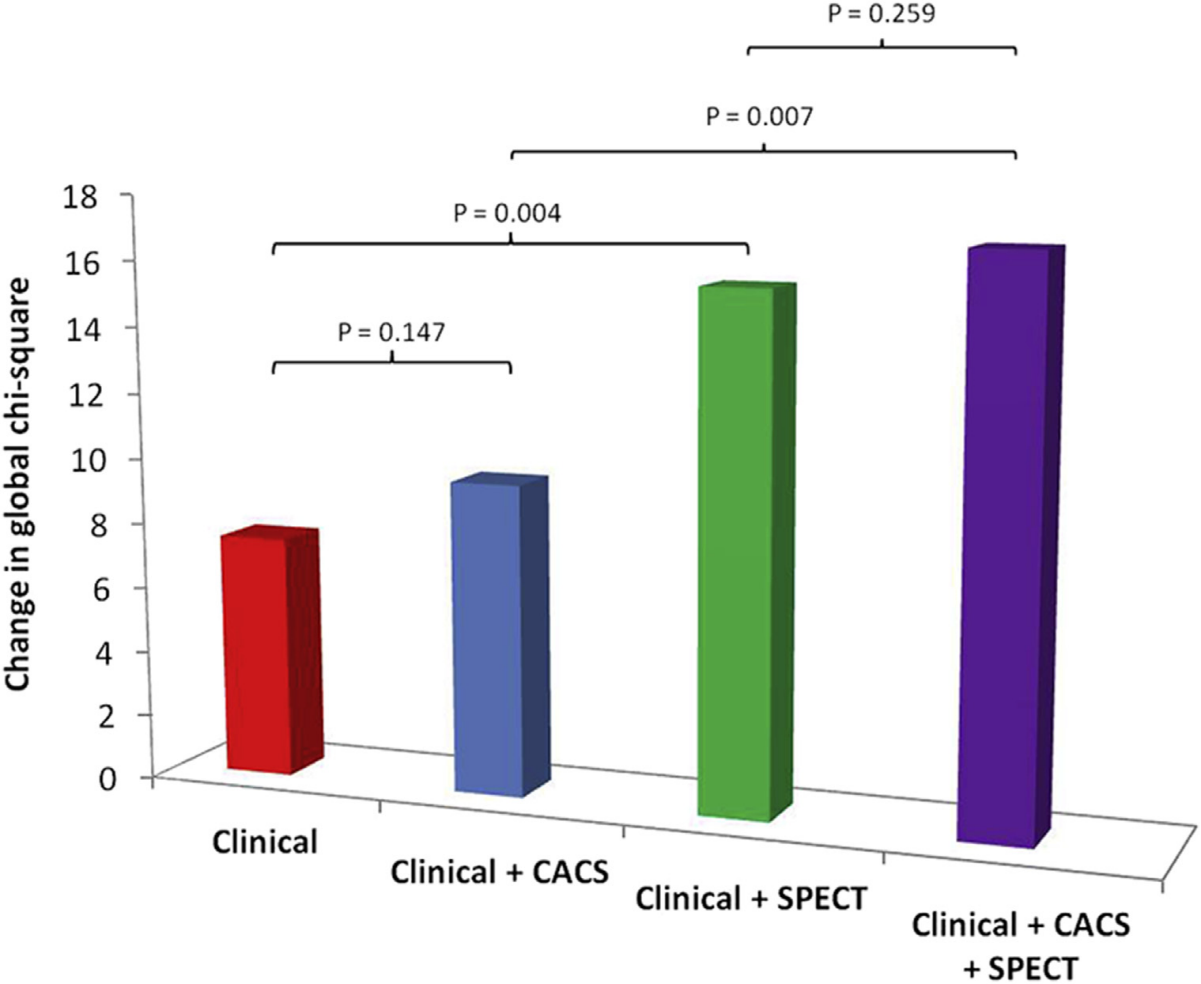
Incremental predictive value of CACS and stress SPECT results over clinical information. The clinical data entered into the global chi-square analysis model included age, gender, and the presence or absence of diabetes. Abnormality on SPECT (defined as SSS >4) and at least moderate calcification (CACS >100 U) were entered as binary variables.

The incremental value of CACS and stress SPECT results to predict the primary event over clinical data by global chi-square analysis is depicted in Figure 6. There was a significant improvement in risk prediction with the addition of abnormal perfusion on SPECT to clinical information (chi-square change = 8.06, p = 0.005). The prognostic value of CACS was not incremental to clinical and SPECT perfusion data (global chi-square change = 2.52, p = 0.112).

## Discussion

This study suggests that quantification of CACS alongside SPECT imaging does not provide incremental prognostic utility for prediction of mortality and nonfatal MI in potential renal transplant candidates. SPECT imaging continued, however, to be a useful method in identifying those subjects with ESRD at high CV risk. In those with abnormal perfusion, the risk for all-cause death/nonfatal MI increased significantly with the presence and extent of SPECT abnormality. Although a CACS >100 U was associated with a worse outcome, the presence of moderate-to-severe CAC did not independently predict outcome after adjusting for clinical data and the SPECT perfusion result. Most patients with ESRD had at least mild coronary calcification (CACS >10 U), but there was a significant proportion (12%) with only minimal CAC who had an abnormal SPECT perfusion result, which continued to confer a higher event rate. This finding demonstrates that the absence of CAC does not eliminate the potential for obstructive CAD in ESRD.

Our study is the first to identify that abnormal perfusion is the more important factor in identifying adverse CV event rates in ESRD relative to the impact of CACS. One previous study in 411 patients with ESRD (86% dialysis dependent) identified a modest association between increasing CACS and abnormal perfusion, as found in our study, but did not examine the association with clinical outcomes.^13^ In general population subjects without advanced CKD, there are conflicting reports regarding the ability of hybrid imaging to predict CV outcomes. Our data are consistent with those of Rozanski et al^26^ which suggest that when perfusion is normal, elevated CACS does not confer an increased risk of CV events. In a further study of 695 consecutive subjects with intermediate risk, abnormal perfusion was associated with adverse CV events even in those subjects with no calcification, albeit with a lower event rate than in those subjects with higher CACS.^27^ However, in a study of 1,126 largely asymptomatic patients, after a much longer duration of follow-up (median 6.9 years), Chang et al were able to demonstrate that CACS offered incremental risk prediction in subjects with a normal perfusion result.^19^ The relative increase in all-cause death/MI was limited to those with CACS >400 U and survival curves only began separating after 3 years, raising the possibility that the impact of CACS on outcome may only be seen after longer follow-up than in our study.

A second possible explanation for the failure of CACS to provide incremental risk predictive value over SPECT in the present study relates to the pathophysiology of arterial calcification in ESRD.^11^ One of the major uses of CACS in the general population has been to identify those at very low risk by confirming the absence of calcification,^28^ but patients with ESRD represent a different challenge. Our study is consistent with others in identifying a remarkably high prevalence of moderate and severe CACS, which may be a consequence of other factors including abnormal calcium–phosphate handling in ESRD rather than reflecting atherosclerosis alone. Indeed, a strong correlation between decreasing glomerular filtration rate and increasing CACS has been demonstrated, such that 3 of 4 subjects with ESRD have a CACS above the 75th centile for gender-and age-matched subjects without ESRD.^29^ Moreover, arterial calcification in ESRD is not limited to the intima (atherosclerosis) causing obstructive coronary disease but also affects the media (arteriosclerosis), which is associated with pressure overload and heart failure.^11^ CACS using 16-slice CT without noninvasive angiography is unable to discriminate between intimal and medial calcification, which may be a further factor contributing to the lack of data associating increasing CACS with an increased CV event rate in ESRD.^11^

There are a number of limitations to our study. These data are from consecutive patients but recruited from a single center with retrospective analysis. The relatively low number of events during follow-up that was limited to a median of 18 months (maximum 3.4 years) may have impacted on our ability to demonstrate an independent association of CACS with hard clinical outcomes. By combining HES with Office for National Statistics data sources, our data linkage process created a complete dataset with regard to mortality. It is possible that events may have been missed for those subjects admitted to hospital abroad, although it would be unusual for patients on renal replacement therapy to leave the country, particularly around the time of work-up for potential transplant. Age did not appear to have a significant influence on adverse outcomes in this cohort. This finding may in part, reflect the relatively narrow age range of our population. There are data that demonstrate traditional CV risk factors are very poor predictors of cardiac events in ESRD.^30^ Annual CV mortality for those receiving maintenance hemodialysis is from 10 to 20 times that of the general population, and younger adults have the greatest increase in CV risk.^1^ Thus, time on maintenance dialysis rather than age may be a more important factor in predicting adverse outcomes, and the lack of data on this variable is an important limitation of our analysis.

## Disclosure

The authors have no conflicts of interest to disclose.

## Figures and Tables

**Table 1:** Baseline demographics and clinical characteristics for study cohort

Variable	n = 194
Age (years)	56.3 ± 10.2
Male	117 (60%)
White	128 (66%)
Asian	49 (25%)
Afro-Caribbean	12 (6%)
Other ethnicity	4 (2%)
Body mass index (kg / m^2^)	27.5 ± 5.0
Diabetes mellitus	64 (33%)
Hypertension∗	159 (82%)
Hypercholesterolemia†	133 (69%)
Current smoker	36 (19%)
Family history of coronary artery disease	38 (20%)
Number of cardiac risk factors	2.3 ± 1.0
Duke pre-test probability (%)	5 (3 – 8)
Symptomatic chest pain	48 (25%)
Typical angina / atypical / non-cardiac	10 (5%) / 20 (10%) / 18 (9%)
Hemoglobin (g/ L)	111 ± 16
Total cholesterol (mg / dL)	185 ± 46
Calcium (mg / dL)	9.00 ± 0.64
Phosphate (mg / dL)	4.30 ± 1.24
Parathyroid hormone, (median pg / mL [IQR])	21.8 (13.1 – 39.9)
Uric acid (mg / dL)	7.13 ± 1.98
CACS (median Agatston units [IQR])	52 (0 – 509)
CACS severity	
0 – 10	68 (35%)
11 – 100	39 (20%)
101 – 400	35 (18%)
>400	52 (27%)
Ability to perform exercise stress	112 (58%)
METS achieved‡	6.7 ± 3.4
Stress electrocardiogram result Normal / Equivocal / Abnormal	130 (67%) / 39 (20%) / 25 (13%)
Left ventricular ejection fraction (median % [IQR])	56 (50 – 62)
Abnormal SPECT§	43 (22%)
Total perfusion deficit score (% LV)	3.9 ± 8.9
Ischemic perfusion deficit score (% LV)	1.6 ± 3.8
Total perfusion deficit score ≥ 15%	18 (9%)
Ischemic perfusion deficit score ≥ 10%	13 (7%)
Medications	
Aspirin	71 (37%)
Thienopyridine	9 (5%)
Beta-blocker	79 (41%)
ACE inhibitor / angiotensin receptor blocker	86 (44%)
Calcium channel blocker	97 (50%)
Loop diuretic	66 (33%)
Statin	123 (63%)
Insulin	42 (22%)

Data are number (%) or mean ± SD unless otherwise stated.

ACE = angiotensin-converting enzyme; CACS = coronary artery calcium score; IQR = interquartile range; LV = left ventricular; METS = metabolic equivalents of task; SPECT = single-photon emission computed tomography.

∗ Defined as an office blood pressure of >140/90 mm Hg or currently taking antihypertensive medications.

† Defined as a fasting serum cholesterol of >193 mg/dl or currently taking lipid reduction therapy.

‡ In the 112 subjects capable of treadmill exercise.

§ Defined as a summed stress score of ≥4.

**Table 2:** Baseline demographics, clinical characteristics, and stress test differences by single-photon emission computed tomography results (n = 194)

Variable	Normal (n = 151)	PDS <15% (n = 25)	PDS ≥15% (n = 18)	p Value∗	IPDS <10% (n = 30)	IPDS ≥10% (n = 13)	p Value†
Age	56.0 ± 10.2	53.9 ± 10.7	62.8 ± 7.3	0.01	54.8 ± 10.0	65.0 ± 8.1	<0.01
Male	88 (58%)	20 (80%)	8 (44%)	0.046	22 (73%)	7 (54%)	0.27
Diabetes mellitus	46 (31%)	15 (60%)	7 (39%)	0.02	10 (33%)	7 (54%)	0.22
Hypertension	124 (82%)	21 (84%)	13 (72%)	0.56	26 (87%)	8 (64%)	0.14
Hypercholesterolemia	104 (69%)	17 (68%)	12 (67%)	0.98	20 (67%)	9 (73%)	0.97
Smoker	71 (47%)	11 (44%)	8 (44%)	0.95	15 (50%)	5 (36%)	0.78
Number of risk factors	2.3 ± 0.1	2.4 ± 1.0	2.4 ± 1.4	0.55	2.4 ± 1.0	2.3 ± 1.5	0.66
Duke pre-test probability (%)	6 (3 – 8)	5 (3 – 8)	7 (3 – 18)	0.01	4 (3 – 7)	10 (4 – 20)	0.02
Symptomatic chest pain	35 (23%)	8 (32%)	5 (28%)	0.61	8 (27%)	5 (36%)	0.46
Ability to perform exercise stress	96 (64%)	11 (44%)	5 (28%)	<0.01	13 (43%)	3 (23%)	<0.01
LV ejection fraction (%)	57 (51 – 63)	55 (50 – 60)	46 (29 – 51)	<0.001	51 (45 – 57)	50 (34 – 61)	<0.001

Data are number (%), mean ± SD or median (interquartile range).

IPDS = ischemic perfusion defect size; PDS = perfusion defect size.

∗ Normal SPECT versus total PDS <15%, total PDS ≥15%.

† Normal SPECT versus ischemic PDS <10%, ischemic PDS ≥10%.

**Table 3:** Baseline demographics, clinical characteristics, and stress test differences by coronary artery calcium score severity

Variable	CACS Severity Groups (n = 194)				
0 – 10 (n = 68)	11 – 100 (n = 39)	101 – 400 (n = 35)	>400 (n = 52)	P Value
Age (years)	51.8 ± 11.5	58.5 ± 7.6	58.1 ± 7.9	59.1 ± 9.6	<0.001
Male	29 (43%)	26 (67%)	21 (60%)	42 (79%)	<0.001
Diabetes mellitus	15 (22%)	12 (31%)	18 (51%)	19 (36%)	0.02
Hypertension	59 (87%)	33 (85%)	26 (74%)	42 (79%)	0.44
Hypercholesterolemia	46 (68%)	25 (64%)	25 (71%)	37 (70%)	0.88
Smoker	29 (43%)	22 (56%)	15 (43%)	24 (45%)	0.55
Number of risk factors	2.2 ± 1.0	2.4 ± 1.0	2.5 ± 1.0	2.3 ± 1.0	0.50
Duke pre-test probability (%)	4 (2 – 5)	5 (4 – 8)	5 (3 – 8)	7 (4 – 9)	0.06
Symptomatic chest pain	24 (35%)	11 (28%)	7 (20%)	8 (15%)	0.07
Ability to perform exercise stress	44 (65%)	23 (59%)	20 (57%)	26 (49%)	0.45
LV ejection fraction (%)	57 (54 – 64)	58 (49 – 62)	57 (48 – 64)	53 (44 – 59)	0.047

Data are number (%), mean ± SD or median (interquartile range).

**Table 4:** Univariate and multivariate predictors of events

Variable	Death or Non-fatal Myocardial Infarction				All-cause Mortality			
Univariate Analysis		Multivariate Analysis		Univariate Analysis		Multivariate Analysis	
HR (95% CI)	P Value	HR (95% CI)	P Value	HR (95% CI)	P Value	HR (95% CI)	P Value
Age	0.99 (0.95 - 1.05)	0.829			0.98 (0.92 - 1.04)	0.463		
Gender (female)	0.85 (0.29 - 2.44)	0.758			0.37 (0.09 - 1.55)	0.173		
Diabetes	3.30 (1.14 - 9.54)	0.028	2.57 (0.87 - 7.59)	0.088	2.46 (0.61 - 9.87)	0.203	1.99 (0.48 - 8.20)	0.339
Current smoker	2.21 (0.74 - 6.62)	0.155			4.34 (1.08 - 17.41)	0.038		
Hypercholesterolemia	0.63 (0.22 - 1.81)	0.390			0.79 (0.19 - 3.31)	0.746		
LV ejection fraction < 55%∗	2.44 (0.84 - 7.05)	0.099			3.20 (0.76 - 13.42)	0.112		
Ability to exercise	0.31 (0.10 - 0.98)	0.046			0.45 (0.11 - 1.89)	0.275		
Abnormal perfusion†	5.32 (1.84 - 15.35)	0.002	4.18 (1.43 - 12.27)	0.009	5.32 (1.84 - 15.35)	0.002	3.00 (0.72 - 12.46)	0.131
At least moderate CACS‡	3.55 (1.11 - 11.35)	0.032	2.50 (0.76 - 8.20)	0.130	2.23 (0.53 - 9.4)	0.273	1.62 (0.37 - 7.13)	0.524

Multivariate regression models were adjusted for age, gender, and diabetes.

∗ Defined by gated single-photon emission computed tomography imaging.

† Defined as summed stress score ≥4.

‡ Defined as coronary artery calcium score >100 U.
